# Serum calcium-magnesium ratio at admission predicts adverse outcomes in patients with acute coronary syndrome

**DOI:** 10.1371/journal.pone.0313352

**Published:** 2024-11-08

**Authors:** Yan Jiang, Baolin Luo, Yaqin Chen, Wen Lu, Yanchun Peng, Liangwan Chen, Yanjuan Lin

**Affiliations:** 1 School of Nursing, Fujian Medical University, Fuzhou, Fujian, China; 2 Department of Nursing, Fujian Medical University Union Hospital, Fuzhou, Fujian, China; 3 Department of Cardiovascular Surgery, Fujian Medical University Union Hospital, Fuzhou, Fujian, China; 4 Fujian Provincial Special Reserve Talents Laboratory, Fuzhou, Fujian, China; Baylor Scott and White, Texas A&M College of Medicine, UNITED STATES OF AMERICA

## Abstract

**Background:**

Evidence from observational studies suggests that increased calcium exposure may elevate the risk of adverse events in patients with coronary artery disease, while magnesium may exert a protective effect on disease risk. However, there have been limited investigations into the relationship between these minerals and acute coronary syndrome (ACS). Therefore, this study aimed to explore the association between the Serum calcium-magnesium ratio (Ca/Mg ratio) in patients with acute coronary syndrome and their clinical outcomes.

**Methods:**

This retrospective analysis reviewed the clinical data of 1,775 patients with ACS who underwent coronary angiography and/or percutaneous coronary intervention (PCI) at the Fujian Heart Center between May 2017 and December 2022. The patients were categorized into four groups based on their Ca/Mg ratio at admission (Group 1, ≤2.373, n = 443; Group 2, 2.374–2.517, n = 442; Group 3, 2.518–2.675, n = 446; Group 4, ≥2.676, n = 444). Single-factor analysis and multivariate logistic regression were employed to analyze the clinical characteristics and postoperative clinical outcomes of patients in different groups. The primary outcome included major adverse cardiovascular and cerebrovascular events (MACCEs), while the secondary outcomes included contrast-induced nephropathy (CIN)、all-cause rehospitalization raten and hematorrhea.

**Results:**

Univariate analysis showed that the patients had a mean age of 64.50±10.79 years, with 370 female patients (20.8%). Additionally, 1,158 patients had hypertension (65.2%), and 710 patients had diabetes (40.5%). Univariate analysis showed an inverse relationship between the serum calcium-to-magnesium ratio and all-cause in-hospital mortality, with patients in the lowest quartile having the highest mortality rate. Multivariate analysis showed that the Ca/Mg ratio at admission was independently associated with MACCEs. Among them, this ratio was inversely associated with all-cause mortality [adjusted odds ratio (aOR) 0.07; 95% CI 0.01–0.63; *P*<0.05] and positively associated with new-onset atrial fibrillation (aOR 1.86; 95% CI 1.08–3.21; *P*<0.05). Additionally, the Ca/Mg ratio was positively correlated with an increased risk of postoperative major bleeding (aOR 6.58; 95% CI 1.43–30.29; *P*<0.05).

**Conclusion:**

In this large retrospective study, serum calcium and magnesium levels at admission were significantly associated with adverse outcomes in patients with ACS. The Ca/Mg ratio was identified as a reliable predictor of poor prognosis in ACS patients.

## Introduction

Globally, the incidence and mortality rates of cardiovascular disease (CVD) are increasing year by year [1]. Acute coronary syndrome (ACS), a critical and severe condition within CVD, is characterized by high urgency and mortality rates. According to the "Blue Book on the Current Status of Acute Coronary Syndrome Prevention and Treatment in China," the in-hospital mortality rate for ACS patients is 5% to 6%, and the recurrence rate of myocardial infarction is 8% to 10% [2]. Percutaneous coronary intervention (PCI) is currently the most commonly used clinical treatment for ACS, significantly improving both the subjective symptoms and objective clinical outcomes of patients. Although PCI can improve survival rates, the risk factors for ACS remain, and patients still face the possibility of coronary restenosis and other cardiovascular events post-surgery [2]. Therefore, addressing the risk factors for cardiovascular disease is crucial to improving the prognosis of ACS patients. Recent studies suggest that the formation and rupture of atherosclerotic (AS) plaques in the coronary arteries are key pathological mechanisms underlying ACS, and this process may be influenced by serum calcium and magnesium levels [3, 4]. The development and progression of coronary atherosclerosis is a chronic, insidious process that begins with endothelial damage in the coronary arteries, followed by abnormal calcium salt deposition. Over time, this accumulation results in calcification. Magnesium, an essential element for maintaining endothelial cell function and a natural calcium antagonist, plays a crucial role in this process. An imbalance in calcium and magnesium ion homeostasis during calcification is a major contributing factor to coronary atherosclerosis and may play a pivotal role in the onset of ACS [5].

ACS is thought to be a clinical condition characterized by the formation of obstructive thrombi due to the rupture or erosion of atherosclerotic plaques. This process activates the coagulation system, with calcium ions playing a key role as a cofactor that is consumed during the cascade. Specifically, lower calcium levels may be associated with increased plaque or thrombus formation, exacerbating disease progression [6]. Serum magnesium, a trace element with significant effects on the cardiovascular system, also plays a crucial role. The relationship between magnesium and atherosclerosis can be summarized as follows: low magnesium levels may elevate lipid concentrations due to endothelial dysfunction, allowing substances like low-density lipoproteins to infiltrate the arterial walls and accumulate. In contrast, higher magnesium levels help reduce lipid levels and maintain normal endothelial function [7]. In summary, serum calcium and magnesium levels can influence the risk of adverse outcomes in patients with ACS. Therefore, maintaining appropriate levels of calcium and magnesium is essential for these patients. Unfortunately, the optimal serum concentrations of magnesium and calcium, as well as the ideal Ca/Mg ratio, remain unresolved.

Since serum calcium and magnesium levels are relatively stable, we collected and analyzed these ions from patients on the first day of admission, before serum electrolytes could be influenced by diuretics or other treatments. This study aims to investigate the relationship between the serum Ca/Mg ratio and the occurrence of adverse clinical outcomes in patients with ACS, while also considering their medical history. By exploring the predictive value of the Ca/Mg ratio in ACS, we seek to assist healthcare professionals in making more accurate and timely clinical decisions, ultimately reducing mortality and readmission rates in ACS patients.

## Materials and methods

### Study design and population

**Fig 1:**
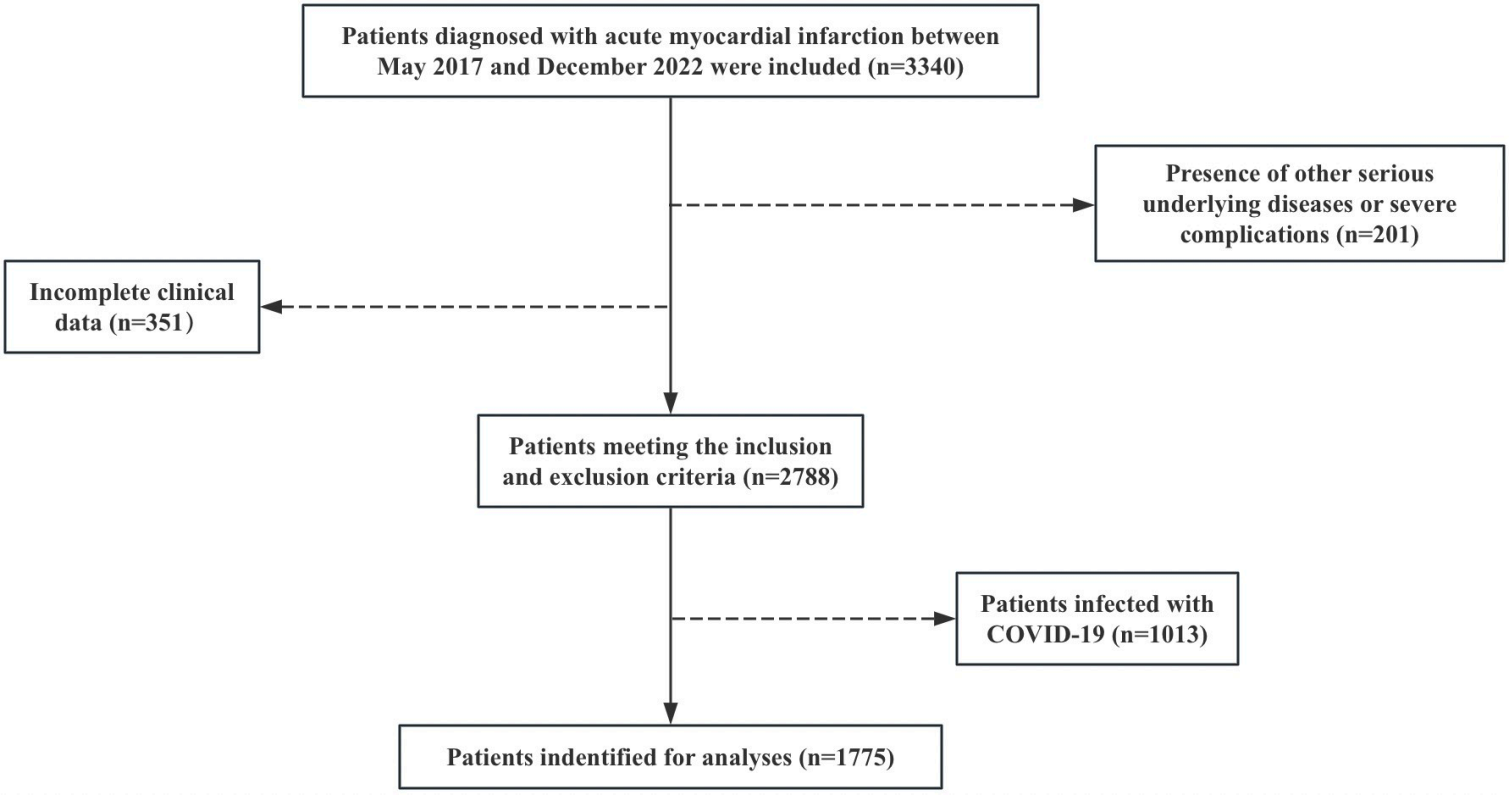
Inclusion/exclusion flowchart for the study group.

This retrospective analysis reviewed the clinical data of 1,775 patients with ACS who underwent coronary angiography and/or percutaneous coronary intervention (PCI) at the Fujian Heart Center between May 2017 and December 2022. The study enrolled patients meeting the following criteria: (1) age≥18 years; (2) all patients met the standards outlined in the Acute Coronary Syndrome Emergency Fast Diagnosis and Treatment Guidelines (2019) published by the Chinese Medical Association Emergency Medicine Branch [8], and excluded those who met the following criteria: (1) coexisting with other severe underlying diseases or serious complications; (2) lacking complete clinical data; (3) Patients infected with COVID-19. Finally, 1,775 patients were included for analysis. Inclusion/exclusion flowchart for the study groups shown in Fig 1.

### Data collection

Venous blood samples were collected within 24 hours of admission. If multiple blood tests were performed within this period, we used the results from the first test. Data on serum calcium and serum magnesium were recorded. These measurements were used to calculate the following biomarker: Ca/Mg ratio = serum calcium/serum magnesium. Baseline data included sociodemographic information, admission and discharge diagnoses, laboratory tests, medications, surgical details, and discharge status.

### Outcomes measured

The primary outcome was the occurrence of major adverse cardiovascular and cerebrovascular events (MACCEs), including all-cause in-hospital mortality, repeat revascularization, new-onset atrial fibrillation (AF) from any cause, stroke, and acute heart failure. All-cause in-hospital mortality was defined as death from any cause during hospitalization. Repeat revascularization was defined as the placement of a second stent during hospitalization, in addition to the initial stent. New-onset atrial fibrillation was defined as atrial fibrillation detected during outpatient or inpatient care using standard electrocardiography, Holter monitoring, or cardiac monitoring devices, with no prior history of atrial fibrillation. Acute heart failure (AHF) refers to the rapid onset or worsening of symptoms and signs secondary to abnormal heart function, accompanied by elevated plasma natriuretic peptide levels. Secondary outcomes included the risk of contrast-induced nephropathy (CIN), major bleeding, and all-cause readmission rates. CIN was defined as a relative increase in serum creatinine concentration by ≥25% or an absolute increase by ≥44.2 μmol/L within 48–72 hours after exposure to contrast agents, excluding other causes of acute kidney injury. Major bleeding included intracranial hemorrhage and upper or lower gastrointestinal bleeding. Any cause of readmission was confirmed by reviewing medical records or contacting the patient or their primary physician. The average follow-up period for this study was one year.

### Statistical analysis

This study aims to investigate the relationship between different serum Ca/Mg ratio levels and clinical outcomes in patients, and to identify the optimal ratio range associated with an increased risk of adverse outcomes. The serum Ca/Mg ratio was divided into four quartiles for analysis (Group 1, ≤2.373, n = 443; Group 2, 2.374–2.517, n = 442; Group 3, 2.518–2.675, n = 446; Group 4, ≥2.676, n = 444). Baseline data for categorical variables were expressed as frequencies and percentages, and group comparisons were analyzed using the χ^2^ test. Continuous variables were assessed for normality using the K-S test, P-P plot, Q-Q plot, and frequency histogram. Normally distributed data were presented as mean±standard deviation and analyzed using ANOVA for group comparisons, while non-normally distributed data were presented as median and interquartile range (IQR) and analyzed using non-parametric tests for group differences. Partial correlation analysis (adjusting for age and gender) was used to evaluate the relationships between serum calcium, magnesium, and the Ca/Mg ratio with apolipoproteins (such as ApoA1, ApoB, and the ApoB/A1 ratio) and lipids (such as TC, TG, LDL-C, and HDL-C). The relationship between baseline Ca/Mg ratio levels and clinical outcomes was assessed using adjusted multivariate logistic regression models. All-cause readmission rates were evaluated using multivariate Cox proportional hazards regression models. Model 1 was adjusted for age and gender, while Model 2 included the variables from Model 1 and other variables with *P*<0.05. Other confounders with a significance level of *P*<0.05 include gender, age, cardiac ejection fraction, history of diabetes, use of calcium channel blockers, ACEI/ARB/ARNI, dual antiplatelet therapy, serum albumin, glomerular filtration rate, blood urea nitrogen, serum creatinine, uric acid, apolipoprotein A, serum chloride, serum calcium, and serum magnesium. Since the variance inflation factor (VIF) for all included variables was <5, indicating no multicollinearity, all variables were included in Model 2 for analysis. Risk levels were evaluated using odds ratios (OR) or hazard ratios (HR) with 95% confidence intervals (CI). *P*<0.05 was considered statistically significant. All statistical analyses were conducted using SPSS version 26.0.

### Ethics approval and informed consent

Ethical approval for this study was granted by the ethics committee of Fujian Medical University Union Hospital (Ref. No. 2023KY032). The committee waived the requirement for informed consent, as the research involved only anonymized patient data collected retrospectively from electronic medical records. Data access for research purposes commenced on May 28, 2023. Throughout the data collection process, the authors had no access to information that could identify individual participants. This study was conducted in accordance with the Declaration of Helsinki, as revised in 2013.

## Results

### Baseline characteristics

This study included 1,775 hospitalized ACS patients with an average age of 64.50±10.79 years, of which 370 were female (20.8%), 1,158 had hypertension (65.2%). Among the patients, the median serum Ca/Mg ratio was 2.52 [P25, P75: 2.37, 2.67]. Based on this ratio, patients were divided into four groups (Group 1, ≤2.373, n = 443; Group 2, 2.374–2.517, n = 442; Group 3, 2.518–2.675, n = 446; Group 4, ≥2.676, n = 444). Patients in Group 4 tended to be younger (*P*<0.05) and exhibited higher left ventricular ejection fractions (*P*<0.05), along with a greater proportion of individuals with diabetes (40.5%, *P*<0.001).

In terms of preoperative medication history, the study found that patients in the fourth quartile of the Ca/Mg ratio used the most medications compared to the other three groups (*P*<0.05). These medications primarily included calcium channel blockers (CCB), antihypertensives (ACEI/ARB/ARNI), and dual antiplatelet therapy (DAPT). Compared to Groups 2, 3, and 4, patients in Group 1, with the lowest Ca/Mg ratio, had significantly higher levels of blood urea nitrogen (*P*<0.05) and serum creatinine (Scr) (*P*<0.05). Notably, these patients also exhibited the highest serum chloride levels (*P*<0.001), which were negatively correlated with the Ca/Mg ratio. In contrast, patients with higher Ca/Mg ratios demonstrated elevated glomerular filtration rates (GFR) and uric acid levels (*P*<0.05), along with an upward trend in serum albumin (ALB) and apolipoprotein A (ApoA) levels (*P*<0.05). More detailed baseline information is provided in Table 1.

**Table 1:** Baseline characteristics in patients with acute coronary syndrome.

	Serum Calcium / Magnesium Ratio Quartile					
Variables	Quartile 1 (≤2.37)	Quartile 2 (2.38–2.52)		Quartile 3 (2.53–2.68)	Quartile 4 (≥2.69)	*P*-value
	(n = 443)	(n = 442)		(n = 446)	(n = 444)	
**Demographic and clinical characteristics**						
Age, years, M (SD)	65.97±10.48	65.29±10.67	63.40±10.85		63.36±10.94	<0.001
Female sex, n (%)	93 (21.0)	83 (18.8)	93 (20.9)		101 (22.7)	0.547
Current smoking, n (%)	176 (39.7)	172 (38.9)	164 (36.8)		170 (38.3)	0.773
Current drinking, n (%)	38 (8.6)	32 (7.2)	35 (7.8)		35 (7.9)	0.952
BMI, kg/m^2^, M (SD)	24.22±3.00	24.46±2.55	24.30±2.50		24.48±3.01	0.445
EF (%), M (SD)	57.58±12.10	59.99±10.88	59.42±10.59		59.92±10.80	0.007
LOS, days, M (SD)	8.63±6.28	8.14±6.76	8.99±6.73		8.66±7.00	0.298
**Complication**						
Hypertension, n (%)	284 (64.1)	290 (65.6)	292(65.5)		292(65.8)	0.952
DM, n (%)	154 (34.8)	152 (34.4)	177(39.7)		236(53.2)	<0.001
Hyperlipemia, n (%)	182 (41.1)	176 (39.8)	191 (42.8)		170 (38.3)	0.562
**Medications before procedures**						
Diuretics, n (%)	85 (19.2)	60 (13.6)	86 (19.3)		80 (18.0)	0.084
CCB, n (%)	97 (21.9)	111 (25.1)	114 (25.6)		136 (30.6)	0.028
ACEI/ARB/ARNI, n (%)	146 (33.0)	134 (30.3)	151 (33.9)		185 (41.7)	0.003
β-blockers, n (%)	235 (53.0)	219 (49.5)	243 (54.5)		249 (56.1)	0.246
Statins, n (%)	345 (77.9)	366 (82.8)	361 (80.9)		374 (84.2)	0.084
DAPT, n (%)	346 (78.1)	372 (84.2)	375 (84.1)		378 (85.1)	0.022
**Procedural character**						
PCI	391 (88.3)	405 (91.6)	393 (88.1)		390 (87.8)	0.229
Number of infarcted arteries,M (SD)	2.76±0.82	2.71±0.83	2.69±0.85		2.76±0.87	0.478
Stent number, M (SD)	1.59±0.89	1.56±0.86	1.60±0.90		1.62±0.90	0.891
**Hepatic Function**						
ALB, g/L, M (SD)	38.33±4.18	39.46±3.91	39.66±4.21		40.10±4.22	<0.001
ALT, IU/L, MED (IQR)	24.00 (16.00,42.00)	23.50 (15.00,37.25)	24.50 (16.00,43.00)		23.00 (15.00,37.00)	0.309
AST, IU/L, MED (IQR)	24.00 (18.00,82.00)	24.00 (18.00,49.00)	25.00 (18.00,62.25)		23.00 (18.00,42.75)	0.055
**Renal Function**						
GFR, mL/min, M (SD)	73.76±18.12	76.03±20.02	77.06±21.04		78.11±23.81	0.010
Urea, mmol/L, M (SD)	6.62±4.36	5.84±2.35	6.08±3.17		5.99±2.43	0.002
Scr,μmol/L, M (SD)	98.11±80.23	84.57±30.98	90.82±72.80		87.10±50.83	0.006
Uric acid,μmol/L, M (SD)	378.16±115.12	379.62±103.50	380.98±100.79		400.07±112.45	0.007
**Blood lipids**						
Total cholesterol, mmol/L, M (SD)	4.19±1.52	4.31±1.19	4.32±1.26		4.32±1.24	0.377
Triglycerides,mmol/L,MED (IQR)	1.38 (1.01,1.87)	1.40 (1.02,1.92)	1.51 (1.07,1.97)		1.43 (1.07,2.13)	0.093
HDL-C, μmol/L, M (SD)	1.01±0.33	1.01±0.24	1.02±0.26		1.04±0.28	0.437
LDL-C, μmol/L, M (SD)	2.72±1.10	2.87±1.10	2.82±1.13		2.74±1.13	0.148
ApoA, g/L, M (SD)	1.17±0.23	1.19±0.21	1.18±0.21		1.21±0.21	0.024
ApoB, g/L, M (SD)	0.89±0.28	0.93±0.28	0.92±0.28		0.91±0.30	0.172
**Serum electrolytes**						
K, mmol/L, M (SD)	4.02±0.45	4.03±0.40	4.02±0.39		4.06±0.45	0.357
Na, mmol/L, M (SD)	140.60±2.93	140.64±2.87	140.32±3.31		140.40±2.78	0.315
Cl, mmol/L, M (SD)	103.26±3.50	103.22±2.98	102.89±3.56		102.19±3.41	<0.001
Ca, mmol/L, M (SD)	2.16±0.11	2.21±0.11	2.24±0.12		2.29±0.11	<0.001
Mg, mmol/L, M (SD)	0.97±0.07	0.90±0.04	0.87±0.04		0.79±0.06	<0.001

Abbreviations: BMI, body mass index; EF, ejection fraction; LOS, length of stay; DM, diabetes mellitus; CCB, Calcium Channel Blockers; ACEI or ARB or ARNI, angiotensin-converting enzyme inhibitors or angiotensin receptor blockers or angiotensin receptor–neprilysin inhibitors; DAPT, dual antiplatelet therapy; PCI, percutaneous coronary intervention; ALB, albumin; ALT, alanine aminotransferase; AST, aspartate aminotransferase; GFR,glomeruar filtration rate; Scr, serum creatinine; HDL-C, High density lipoprotein cholesterol; LDL-C, Low-Density Lipoprotein Cholesterol; ApoA, Apolipoproteins A; ApoB, Apolipoproteins B

### Relationship between Ca/Mg ratio and other serum electrolytes

Spearman correlation analysis revealed a significant negative correlation between the Ca/Mg ratio and serum chloride levels (r = -0.831, *P*<0.001). However, there was no significant correlation with serum potassium (r = 0.040, *P* = 0.088) or sodium (r = -0.038, *P* = 0.112).

### Ca/Mg ratio as a predictor of clinical outcome

In the entire study population, the incidence of adverse outcomes was relatively high. Different Ca/Mg ratios at admission were independently associated with postoperative MACCEs, including All-cause in-hospital mortality, new-onset atrial fibrillation, and postoperative major bleeding in ACS patients (*P*<0.05).

**Fig 2:**
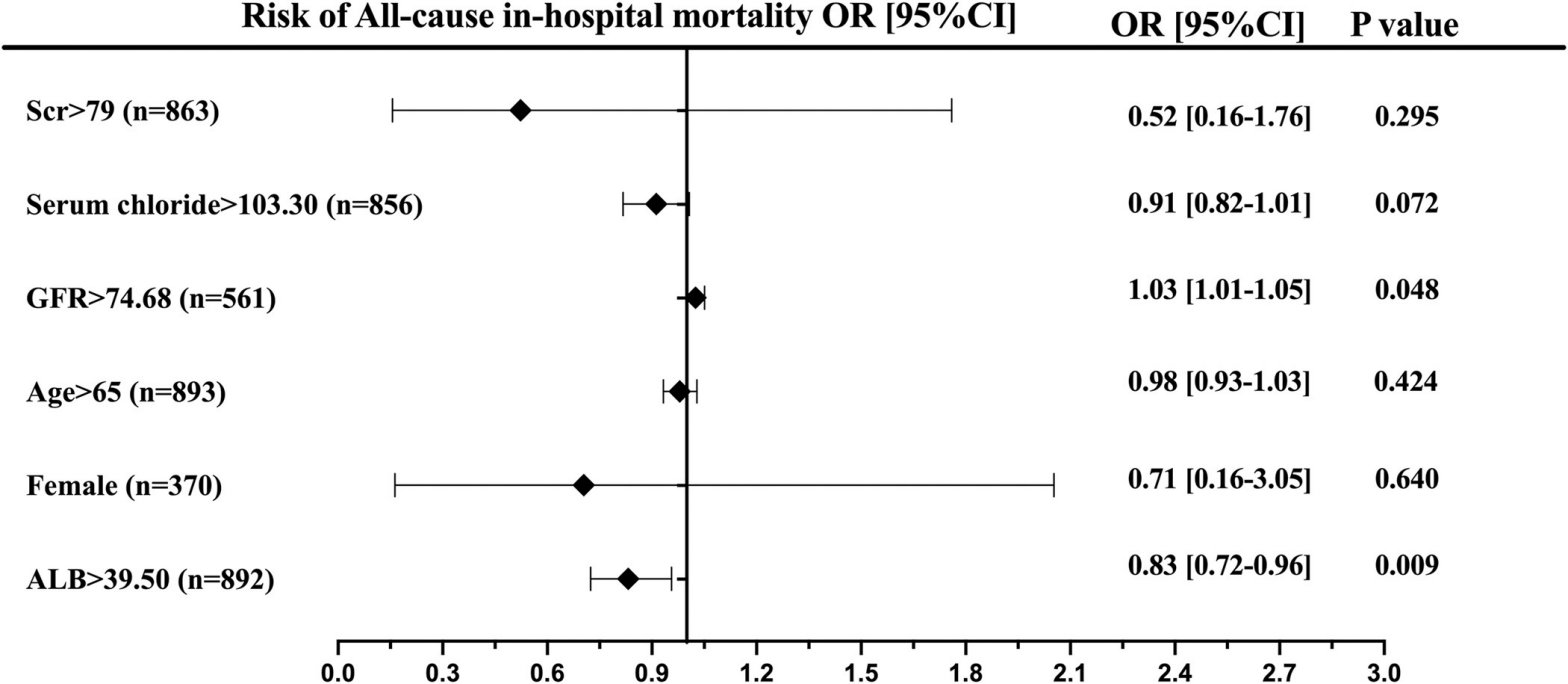
Forest plot for the effects sizes of individual predictors of all-cause in hospital mortality in patients with acute coronary syndrome. Abbreviations: Scr, serum creatinine; GFR,glomeruar filtration rate; ALB, albumin.

A significant negative correlation was found between the Ca/Mg ratio and all-cause in-hospital mortality (3.4% vs. 1.1% vs. 1.1% vs. 0.5%, *P*<0.05), primarily driven by the lowest quartile level. This result was further confirmed after adjusting for baseline confounding factors in Model 2 [adjusted odds ratio (aOR) 0.07; 95% CI 0.01–0.63; *P*<0.05). Subgroup analysis revealed that ALB>39.50g/L was a protective factor against mortality risk (*P*<0.05) (Fig 2) (Table 2).

**Table 2:** The risk of clinical outcomes in patients with acute coronary syndrome.

		Serum Calcium / Magnesium Ratio Quartile				
		Quartile 1 (≤2.37)	Quartile 2 (2.38–2.52)	Quartile 3 (2.53–2.68)	Quartile 4 (≥2.69)	value
Main outcomes	Model	(n = 443)	(n = 442)	(n = 446)	(n = 444)	
**MACCEs**						
All-cause in-hospital mortality	All-cause in-hospital mortality	15 (3.4)	5 (1.1)	5 (1.1)	2 (0.5)	0.002
	Non-All-cause in-hospital mortality	428 (96.6)	437 (98.9)	441 (98.9)	442 (99.5)	NA
	Model 1	Reference	0.32 (0.12,0.90)	0.31 (0.11,0.88)	0.13(0.03,0.55)	0.006
	Model 2	Reference	0.26 (0.07,0.95)	0.15 (0.03,0.71)	0.07(0.01,0.63)	0.048
Repeated revascularization	Repeated revascularization	45 (10.2)	35 (7.9)	37 (8.3)	32 (7.2)	0.433
	Non-Repeated revascularization	398 (89.8)	407 (92.1)	409 (91.7)	412 (92.8)	NA
	Model 1	Reference	0.76 (0.48,1.21)	0.79 (0.50,1.25)	0.68(0.42,1.09)	0.413
	Model 2	Reference	0.67 (0.37,1.19)	0.66 (0.32,1.33)	0.51(0.19,1.43)	0.549
Atrial fibrillation	AF	36 (8.1)	25 (5.7)	35 (7.8)	40 (9.0)	0.283
	Non-AF	407 (91.9)	417 (94.3)	411 (92.2)	404 (91.0)	NA
	Model 1	Reference	0.70 (0.41,1.19)	1.15 (0.70,1.89)	1.36(0.84,2.20)	0.096
	Model 2	Reference	0.85 (0.48,1.49)	1.38 (0.81,2.35)	1.86(1.08,3.21)	0.032
Stroke	Stroke	52 (11.7)	45 (10.2)	40 (9.0)	53 (11.9)	0.435
	Non-Stroke	391 (88.3)	397 (89.8)	406 (91.0)	391 (88.1)	NA
	Model 1	Reference	0.86 (0.56,1.32)	0.82 (0.53,1.27)	1.14(0.76,1.73)	0.419
	Model 2	Reference	0.93 (0.57,1.50)	0.90 (0.52,1.55)	1.26(0.64,2.48)	0.532
Acute heart failure	Acute heart failure	4 (0.9)	3 (0.7)	2 (0.4)	1 (0.2)	0.567
	Non-Acute heart failure	439 (99.1)	439 (99.3)	444 (99.6)	443 (99.8)	NA
	Model 1	Reference	0.74 (0.16,3.31)	0.48 (0.09,2.67)	0.25(0.03,2.22)	0.602
	Model 2	Reference	1.12(0.05,22.98)	0.22 (0.01,8.31)	0.01(0.00,6.70)	0.305
**Secondary outcomes**						
CIN	CIN	22 (5.0)	18 (4.1)	27 (6.1)	30 (6.8)	0.307
	Non-CIN	421 (95.0)	424 (95.9)	419 (93.9)	414 (93.2)	NA
	Model 1	Reference	0.74 (0.16,3.31)	0.48 (0.09,2.67)	0.25(0.03,2.22)	0.602
	Model 2	Reference	0.80 (0.38,1.70)	0.77 (0.32,1.85)	0.81(0.24,2.68)	0.924
All-cause readmission	All-cause readmission	155 (35.0)	144 (32.6)	146 (32.7)	141 (31.8)	0.765
	Non-All-cause readmission	288 (65.0)	298 (67.4)	300 (67.3)	303 (68.2)	NA
	Model 1	Reference	0.98 (0.78,1.24)	0.98 (0.78,1.23)	1.03(0.82,1.29)	0.978
	Model 2	Reference	1.10 (0.83,1.47)	1.08 (0.77,1.54)	1.16(0.71,1.89)	0.919
Hematorrhea	Hematorrhea	14 (3.2)	15 (3.4)	19 (4.3)	13 (2.9)	0.714
	Non-Hematorrhea	429 (96.8)	427 (96.6)	427 (95.7)	431 (97.1)	NA
	Model 1	Reference	1.08 (0.52,2.27)	1.45 (0.71,2.94)	0.99 (0.46,2.13)	0.667
	Model 2	Reference	2.54 (0.95,6.79)	4.94(1.60,15.31)	6.58(1.43,10.29)	0.048

Abbreviations: MACCEs, major adverse cardiovascular and cerebrovascular events; CIN, contrast-induced nephropathy; Model 1 was adjusted for age and sex; Model 2 was adjusted for Model 1 plus other risk factors, other risk factors include gender, age, cardiac ejection fraction, history of diabetes, use of calcium channel blockers, ACEI/ARB/ARNI, dual antiplatelet therapy, serum albumin, glomerular filtration rate, blood urea nitrogen, serum creatinine, uric acid, apolipoprotein A, serum chloride, serum calcium, and serum magnesium.

**Fig 3:**
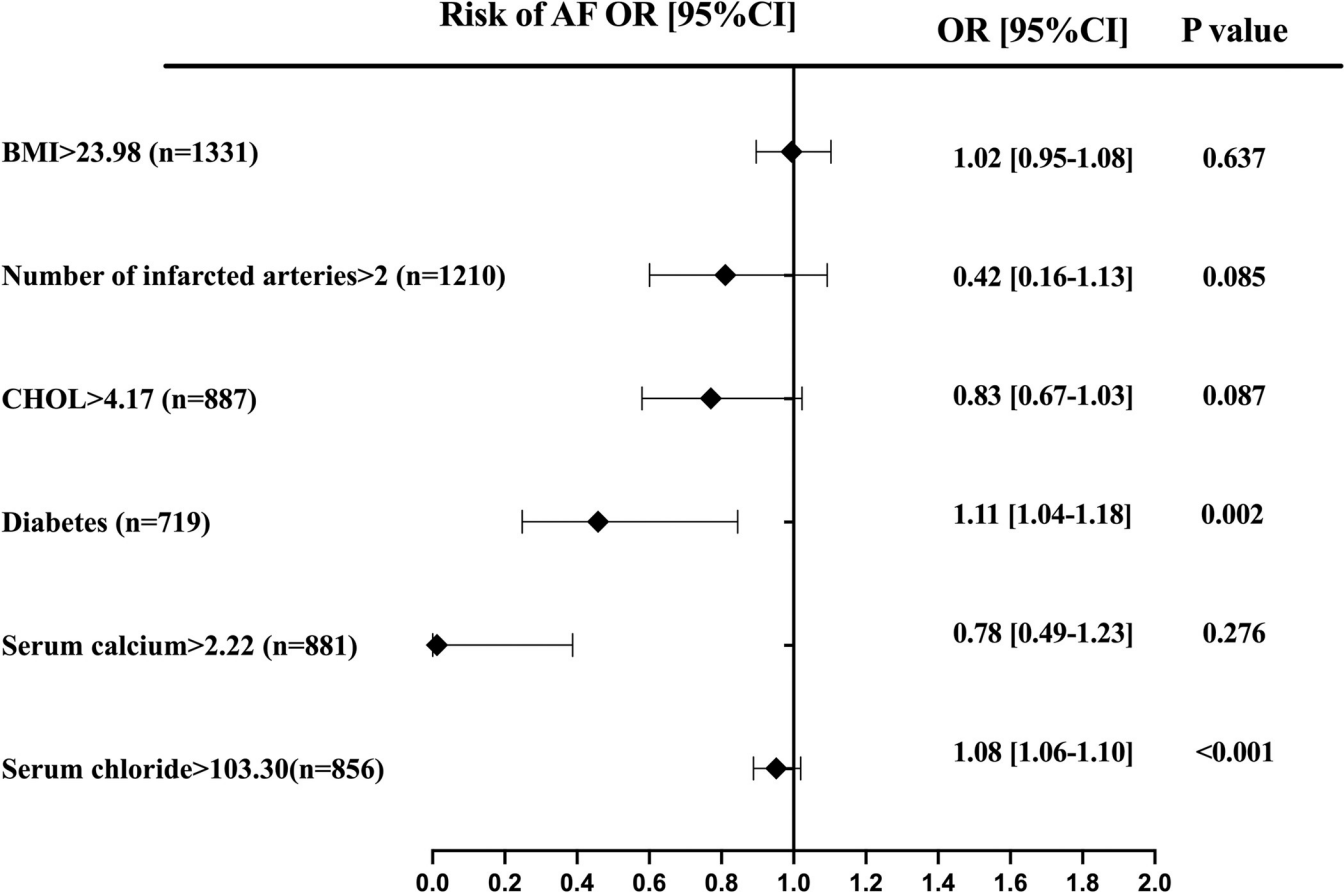
Predictors of atrial fibrillation in patients with acute coronary syndrome. Forest plot for the effects sizes of individual predictors of atrial fibrillation in patients with acute coronary syndrome. Abbreviations: AF, atrial fibrillation; ALB, albumin; ApoA, Apolipoproteins A.

Similarly, there was no difference in the incidence of new-onset atrial fibrillation among patients with different Ca/Mg ratio levels at admission (*P*>0.05). However, after controlling for confounding factors, multivariable analysis showed statistically significant differences between groups (aOR 1.86; 95% CI 1.08–3.21; *P*<0.05). Subgroup analysis identified urea levels greater than 5.40 mmol/L and age over 65 years as independent risk factors for atrial fibrillation (*P*<0.05) (Fig 3) (Table 2).

**Fig 4:**
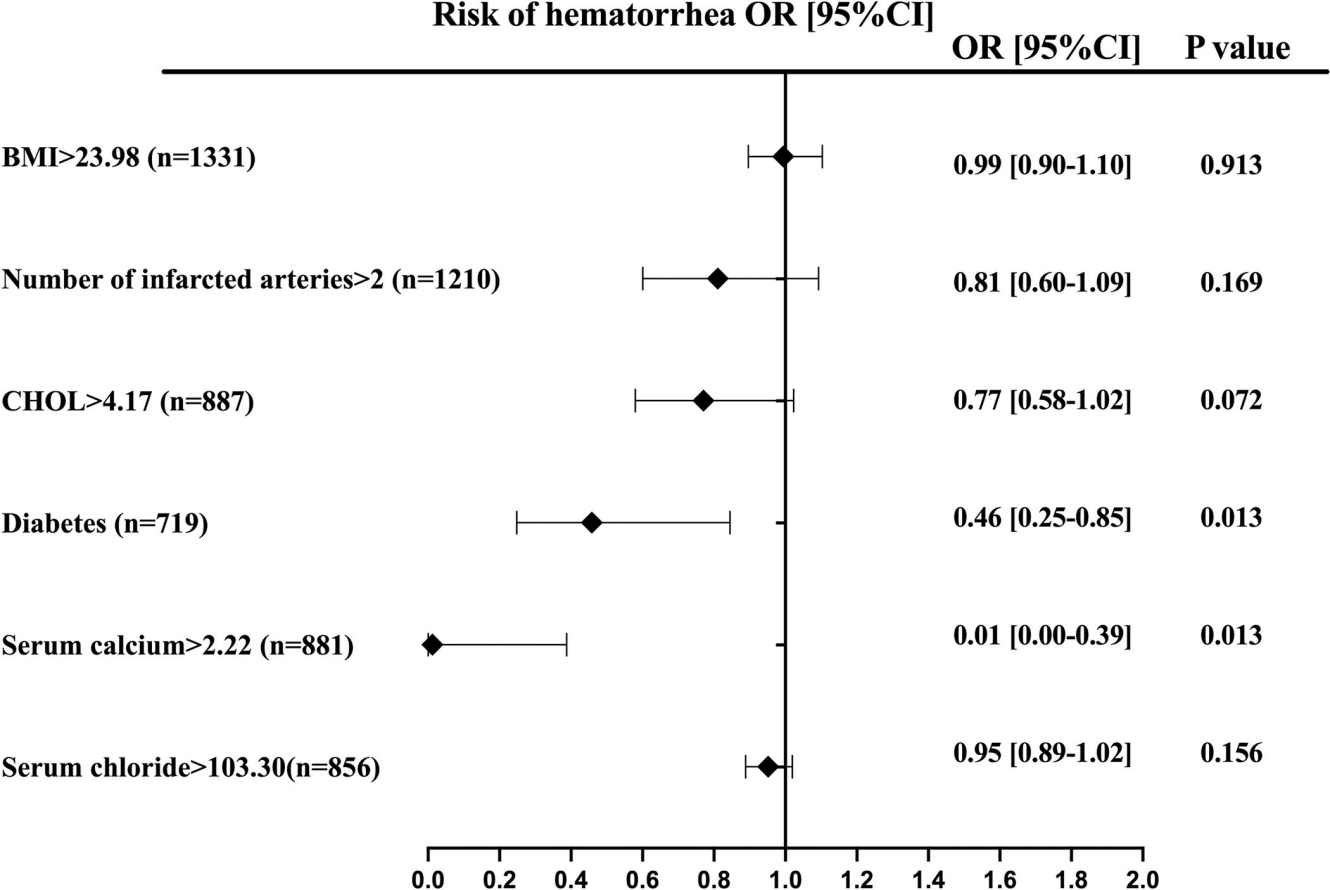
Predictors of hematorrhea in patients with acute coronary syndrome. Forest plot for the effects sizes of individual predictors of hematorrhea in patients with acute coronary syndrome. Abbreviations: BMI, body mass index; CHOL, total cholesterol.

Additionally, we found that the incidence of major bleeding in this study population was 3.4%. Patients with the lowest quartile Ca/Mg ratio at admission had the lowest risk of major bleeding, although the difference between groups was not statistically significant (2.9%, *P*>0.05). Multivariable analysis, however, indicated statistically significant differences between groups (aOR 6.58; 95% CI 1.43–10.29; *P*<0.05). Notably, subgroup analysis of high-risk patients showed that diabetes was a risk factor for major bleeding (*P*<0.05), whereas serum chlorine>2.22mmol/L inhibited major bleeding outcomes (*P*<0.05) (Fig 4) (Table 2).

Finally, even after fully adjusting for confounding factors, there was no significant difference in the risk of repeat revascularization, stroke, acute heart failure, CIN, or all-cause readmission rates among the groups (*P*>0.05) (Table 2).

## Discussion

Currently, the relationship between the Ca/Mg ratio and cardiovascular mortality remains inconclusive. To our knowledge, this study is the first to investigate the relationship between the Ca/Mg ratio and clinical outcomes in patients with ACS who underwent coronary angiography and/or PCI. ACS is hypothesized to be a clinical condition characterized by either complete or partial thrombotic occlusion, resulting from the rupture or erosion of atherosclerotic plaques. This process triggers the coagulation system, with calcium ions playing a crucial role as a cofactor, consumed during the cascade [6]. Serum magnesium, an essential element for the human body. The relationship between magnesium levels and atherosclerosis can be summarized as follows: low magnesium concentrations may elevate lipid levels, whereas higher magnesium levels help lower lipid levels, enhance endothelial function, and stimulate nitric oxide synthesis, thereby maintaining normal endothelial function [7].

Notably, the high mortality associated with ACS is partly attributed to increased calcification of the intimal layer of large arteries, including the aorta. This aortic calcification contributes to arterial stiffness, elevated pulse pressure, and reduced diastolic myocardial perfusion, ultimately leading to ischemia and patient mortality [9]. Consequently, an imbalance in calcium and phosphorus metabolism can result in passive calcium deposition in tissues, causing vascular calcification. As a natural calcium antagonist, magnesium plays a crucial role; thus, its imbalance during the calcification process is a significant factor contributing to coronary calcification and potentially accelerated mortality in ACS patients [5]. Our findings indicate that patients in the lowest quartile of the Ca/Mg ratio had the highest in-hospital all-cause mortality. This association remained significant even after adjusting for baseline confounding factors, consistent with the results of several other studies [10, 11]. This suggests that all-cause mortality decreases with increasing Ca/Mg ratio. In other words, lower serum calcium levels and higher magnesium levels are associated with increased mortality risk. Additionally, previous studies have shown a strong association between serum albumin levels and in-hospital mortality [12, 13]. The findings of this study confirm this relationship. Subgroup analysis revealed that albumin levels above 39.50 g/L serve as a protective factor against mortality risk, consistent with the results of other studies. As a traditional serum marker, albumin reflects nutritional status, which is closely related to malnutrition and significantly correlated with mortality risk in ACS patients. This underscores the importance of monitoring high-risk populations.

Current mainstream views suggest that electrolyte imbalances are associated with the onset of new AF [14]. Serum calcium, an important electrolyte, significantly impacts cardiac electrical activity and contractility [15]. Evidence indicates that hypercalcemia can reduce cardiac conduction velocity and shorten the refractory period, potentially facilitating reentrant mechanisms and complex ventricular arrhythmias such as AF [16]. Magnesium, acting as a calcium antagonist, regulates myocardial excitability by inhibiting calcium entry into cells, thereby exhibiting antiarrhythmic effects [17]. A small study suggested that restricting dietary magnesium intake to less than half the recommended daily allowance increases the risk of supraventricular ectopy and AF, supporting the link between hypomagnesemia and arrhythmias [18]. In our study, the Ca/Mg ratio was closely related to the risk of AF; specifically, patients in the highest quartile of this ratio exhibited the highest incidence of AF, aligning with findings from other research [19]. Subgroup analysis of high-risk patients further revealed that age is the most prominent risk factor for the onset, prevalence, and progression of AF. Studies have shown that elderly AF patients have significantly elevated levels of HCN2 and HCN4 channels in the right atrium, further triggering AF [20]. Additionally, we found that increased blood urea nitrogen levels were also associated with a higher risk of AF, indicating that electrolyte imbalances caused by renal dysfunction may precipitate AF [21]. This highlights the need for healthcare providers to closely monitor patients with renal insufficiency.

The dynamic effects and mechanisms of serum calcium and magnesium on massive hemorrhage in patients with ACS warrant further investigation. Current research has established that calcium ions play a crucial role in platelet aggregation and the coagulation cascade, thereby contributing to hemostasis. Additionally, calcium serves as a cofactor within enzyme systems, influencing vasomotor tone control and significantly affecting the contractility of both myocardial and striated muscle [22, 23]. An in vitro study has demonstrated a correlation between ionic calcium levels and clot strength as well as concentration [24]. Regarding the relationship between serum magnesium levels and bleeding tendencies, Steffen et al. have shown that magnesium can interfere with intracellular Ca^2+^ mobilization, subsequently impacting platelet aggregation [25]. Furthermore, it inhibits platelet activation by obstructing thromboxane A2 production and interfering with the IIb-IIIa receptor complex [26]. In summary, the combined effects of serum calcium and magnesium render patients particularly susceptible to coagulopathy. It is widely recognized that individuals with coagulopathy are at an increased risk for various types of bleeding events. Notably, statistical analyses from this study revealed that higher serum Ca/Mg ratios upon admission—especially within the second and third quartiles—were significantly associated with an elevated risk of major bleeding among ACS patients. This association remained significant following additional multivariate analysis. To date, no researchers have explored how adjusting the serum calcium/magnesium ratio may influence bleeding outcomes in ACS patients; thus, further research is essential in this domain. Further subgroup analysis of the data indicated that diabetes (DM) is a significant risk factor for major bleeding in patients with ACS. This association may be attributed to the reduced levels of superoxide dismutase observed in ACS patients with diabetes, which leads to heightened oxidative stress and altered expression of vascular endothelial growth factor receptors. Consequently, this increases the susceptibility of endothelial cells to major bleeding events [27]. Moreover, hyperglycemia and insulin resistance can exacerbate platelet aggregation and smooth muscle contraction, despite a decrease in the functionality of highly activated platelets. Patients with diabetes exhibit a higher incidence of thrombosis, restenosis, and bleeding complications following PCI compared to their non-diabetic counterparts [28]. Therefore, it is clinically imperative to conduct preoperative assessments of serum calcium and magnesium levels, accurately evaluate bleeding risks in ACS patients, identify high-risk groups, and appropriately adjust treatment regimens—including antithrombotic combination therapies.

## Limitations

Our study, the largest to date and the first to explore the association between different levels of the Ca/Mg ratio and the prognosis of patients with acute coronary syndrome, provides valuable evidence for improving clinical outcomes. However, there are several limitations. Firstly, as a single-center retrospective study, confounding factors are inevitable. Secondly, the Ca/Mg ratio was calculated based on blood biochemical tests at admission, without dynamic monitoring. Since the optimal time for sample collection is unclear, future research should focus on monitoring these indicators dynamically throughout the entire treatment process, rather than relying solely on baseline measurements at admission. Lastly, the follow-up period in this study was relatively short, with an average follow-up time of 1 year, lacking long-term outpatient follow-up. In future studies, we plan to extend the longitudinal follow-up period and include more comprehensive outpatient outcomes.

## Conclusion

In our retrospective study, the Ca/Mg ratio emerged as a strong predictive indicator of adverse outcomes in cardiovascular disease patients, particularly in those with ACS. Healthcare professionals should pay close attention to the concentrations of calcium and magnesium ions in the blood of ACS patients, as this information can provide valuable insights for early intervention in high-risk cases. However, we recommend that future longitudinal studies be conducted to obtain more reliable evidence regarding the roles of these serum electrolytes, specifically serum calcium and magnesium, in the context of ACS.
